# The act of telling: South African women’s narratives of HIV status disclosure to intimate partners in the HPTN 071 (PopART) HIV prevention trial

**DOI:** 10.1177/1745506521998204

**Published:** 2021-02-27

**Authors:** Lario Viljoen, Dillon Wademan, Graeme Hoddinott, Virginia Bond, Janet Seeley, Peter Bock, Sarah Fidler, Lindsey Reynolds, on behalf of the HPTN 071 (PopART) team

**Affiliations:** 1 Desmond Tutu TB Centre, Department of Paediatrics and Child Health, Faculty of Medicine and Health Sciences, Stellenbosch University, Stellenbosch, South Africa; 2 Department of Sociology and Social Anthropology, Stellenbosch University, Stellenbosch, South Africa; 3 School of Public Health, Zambart, University of Zambia, Lusaka, Zambia; 4 Department of Global Health and Development, Faculty of Public Health and Policy, London School of Hygiene and Tropical Medicine, London, UK; 5 Imperial College NIHR BRC, Imperial College London, London, UK

**Keywords:** disclosure, HIV, performance, relationships, universal HIV testing and treatment

## Abstract

**Background:**

Public health programming often frames HIV status disclosure as a means to negotiate condom- and abstinence-based prevention or to involve intimate partners in HIV care to garner treatment adherence support. HIV treatment can be used to ensure viral suppression and prevent onward transmission, which provides strong evidence to encourage disclosure. The ideological shift towards HIV treatment as prevention is expected to facilitate disclosure.

**Purpose:**

There is a lack of research on how the scale-up of universal HIV testing and treatment influences disclosure practices in high burden settings. In this manuscript, we aim to address this gap.

**Methods:**

To this end, we conducted a two-phased narrative performative analysis of the disclosure scripts of 15 women living with HIV in three communities of Western Cape, South Africa where the HPTN 071 (PopART) HIV prevention trial implemented a universal HIV testing and treatment model as part of the intervention. The women were part of a larger cohort nested in the trial. We use Goffman’s dramaturgical metaphor, which understands social interactions as ‘performances’ by ‘actors’ (people) guided by ‘scripts’ (anticipated dialogues/interactions), to explore how women living with HIV manage their status disclosure.

**Conclusion:**

We describe how these women perform HIV status disclosure (or deliberate non-disclosure) to retain, reaffirm or redefine existing social scripts with partners. Their performances reveal priorities other than those imagined by public health programmes driving HIV disclosure (or non-disclosure): establishing trust, resenting betrayal and ensuring self-preservation while simultaneously (re)constructing self-identity. None of the women engaged with the concept of treatment as prevention in their disclosure narratives, either to facilitate disclosure or to ‘justify’ non-disclosure. HIV prevention, in general, and treatment adherence support were rarely mentioned as a reason for disclosure. To date, there has been a missed opportunity to ease and support disclosure in health programmes by tapping into existing social scripts, impeding potential patient and public health benefits of universal HIV testing and treatment.

## Introduction

In South Africa, voluntary status disclosure is framed as an individual and social good in HIV prevention and treatment programmes and is often encouraged by health workers as an important first step after diagnosis.^1,2^ Status disclosure to intimate sexual partners is usually positioned by health implementers as a way to either negotiate condom- or abstinence-based methods to prevent onward HIV transmission^3,4^ or to ensure antiretroviral therapy (ART) adherence support.^5,6^

Globally, there have been efforts to scale-up access to ART (WHO 2018)^7^ as ART holds both individual’s health benefits^8^ and has the ability to prevent onward HIV transmission when people living with HIV (PLHIV) are virally suppressed.^9^ Health providers often position HIV status disclosure as a catalyst for treatment access and adherence through increased social support and as a means to prevent transmission when sexual partners are able to make informed decisions around safe sex.^1,2,5^ With the established health benefits of ART and the ability to prevent onward transmission, the diverging reasons for disclosure are brought closer together. Evidence from health settings in South Africa has shown that the message of treatment as a means of preventing HIV transmission has not being explicitly advocated, and authors have noted that it will be difficult to implement and will require appropriate resources at every step in the HIV care continuum.^10,11^ In addition, research from trial settings has shown that despite ART availability to all PLHIV, ART coverage is undermined by suboptimal linkage to care.^12,13^ We suggest that messages of HIV treatment as a form of prevention might ease and facilitate more disclosure among PLHIV in intimate relationships and encourage linkage to care. However, there is a lack of empirical data on how, or if, the offer of universal access to testing and treatment (UTT) and messages surrounding treatment as prevention are filtering into the disclosure practices of people in high burden settings. To understand the motivations behind and pathways of HIV disclosure in the context of UTT, we conducted a performative analysis^14^ of the narratives of women living with HIV in South Africa, the country with the most PLHIV.^15^

For PLHIV, a positive diagnosis has both biological and social implications. HIV has been described as ‘an epidemic of signification’^16^ that has to be managed ‘both clinically and socially’.^17^ The historical association of HIV with sex, promiscuity and death^18^ means that it is a highly stigmatized condition^19,20^ and that for many PLHIV, their diagnosis is positioned as a private condition.^17,21,22^ As is the case for other chronic illnesses, the act of disclosure (or deliberate non-disclosure) often means more than just revealing health information, but rather, signifies the performance of broader underlying interpersonal processes and the reconciling of self and identity,^23^ where ‘self’ can be understood as the sense of who one is in relation to others.^24^ The aim of this article is to explore how women living with HIV negotiate disclosure (or non-disclosure) of their HIV status with their sexual partners through social scripts and performances.

### HIV status disclosure: health and social consequences

Studies have found that HIV status disclosure is associated with an increased likelihood of ART initiation and adherence,^5,25^ increased social support^26^ and safer sexual practices among intimate partners.^27,28^ As a result, status disclosure is generally regarded as an important part of HIV treatment adherence and is actively promoted in many settings.^2^ As HIV is transmitted mainly through sex in South Africa,^29^ researchers need to focus on whether individuals decide to disclose in sexual relationships, or not. HIV status disclosure in intimate sexual relationships is subject to various dynamics, including perceived relationship quality and type, decisions around childbearing, knowledge of others who have disclosed their status, HIV knowledge and individuals’ self-esteem.^27,30,31^

Disclosure is further complicated by associations with enacted stigma,^32^ while non-disclosure is associated with internalized and anticipated stigma.^33^ Intertwined with these factors are broader social processes, including gender roles and power dynamics.^34,35^ For example, fears around intimate partner violence^17^ render disclosure problematic. Abdool Karim et al.^36^ found that lower rates of HIV status disclosure by women to their sexual partners are ‘suggestive of significant gender-power imbalance within couples . . . [highlighting the] fear of enacted stigma and gender-based violence as significant barriers to disclosure’. Against this background, we explore how South African women in heterosexual relationships ‘perform’ HIV status disclosure (or non-disclosure).

### Performance and disclosure

According to Goffman,^37^ social interactions between people, including health status disclosures, are similar to theatrical performances. Goffman employs stage metaphors of actors and audiences (participants in social interactions); roles and scripts (anticipated dialogues and interactions); the front stage (where persons ‘do’ performances for others) and the backstage (where individuals retreat from their public, social performances) to understand how persons operate and sustain accepted/normative social interactions or scenarios. Goffman highlights how performances are key in constructing and maintaining self-identity. Building on Goffman’s work, Brissett and Edgley^38^ noted that the self is ‘established by its activity and the activity of others with respect to it . . . [and] selves are outcomes, not antecedents, of human interaction’. Performances, in addition to being mechanisms of maintaining accepted social scripts, are thus instrumental in constructing identity. Goffman noted that people are constantly negotiating impression management whereby, to preserve positive self-identities, individuals attempt to shape favourable impressions of themselves and avoid embarrassment or social discomfort.

In relation to health conditions, Bury^23^ noted that illness, especially a chronic illness, can be understood as a ‘biological disruption’, as it upends the structures of everyday life. By carefully crafting how, when and to whom they disclose their status, people with chronic illnesses create ‘bounded impressions’ or ‘scripts’ of themselves through which they attempt to regulate others’ reception of the information and maintain a positive identity.^39^ Interactions are, therefore, structured around actors’ mutual understandings of the accepted ‘scripts’ for social scenarios. When people disclose their illness status, it is feasible that they want to convey a certain message (or performance) and hold the expectation that their partner will adhere to an anticipated script or react in predictable ways.^39^

The public health ‘script’ for HIV status disclosure has focused mainly on the so-called ‘logico-scientific reasoning’ approach, whereby inferences are made from pragmatic empirical evidence as opposed to an intuitive narratives approach, where subjective experiences form the basis of knowledge.^40^ Accordingly, HIV status disclosure is framed as a means of transferring knowledge of a diagnosis to make informed, and by implication, better future (sexual) health decisions. However, research has shown that HIV status disclosure has repercussions for intimate partners that PLHIV consider when deciding if, why and how to share their status. HIV status disclosure is related to the anticipated reactions from significant others^41^ and has been linked to strengthening identity and intimate relationships,^42^ while non-disclosure has been linked to fear, mistrust and anticipated stigma.^35,41^ We explore how HIV status disclosure is framed by women in South Africa at this stage of the HIV epidemic where treatment is available for all PLHIV.

## Methods

### Context

The data for this analysis were drawn from the social science component of a cluster-randomized HIV prevention trial referred to as HPTN 071 (or PopART).^43^ Through a cadre of community-based health workers, community members were offered door-to-door testing and a package of HIV prevention interventions consisting of condoms, STI screenings, referrals for medical male circumcision, and, in communities randomized to receive the full intervention, early access to ART to PLHIV (ART regardless of CD4 count). The aim of the trial was to measure the impact of universal access to HIV testing and treatment on HIV incidence. The trial was conducted in 9 communities in the Western Cape Province of South Africa and 12 communities across Zambia. This analysis is based on the data we collected with 89 households participating in a longitudinal qualitative cohort study nested in the 9 trial communities in South Africa. Households were recruited through referrals by community health workers and during structured community observations.

We conducted bimonthly household visits over an 18-month period between 2016 and 2018. Researchers worked in pairs, usually a man and a woman, with at least one researcher fluent in the participants’ home language. We used participatory research methods and completed thematized discussion guides arranged by modules with family members, either as one-on-one interviews or as group discussions, depending on the topic and household structure. Modules focused on household composition; income and expenditure; love, sex and romance; health access; and hopes, dreams and fears. Households were recruited to ensure diversity across age, gender, composition and HIV status (including participants who self-reported as living with or not living with HIV).^21^

### Sample

For this analysis, we included all cisgender women living with HIV in the three South African intervention communities receiving early access to ART and who participated in the qualitative cohort (n = 15) (Transgender women were excluded from the analysis as they have unique, intersecting experiences of HIV risk, status disclosure and relationship dynamics.^44^).

### Analysis

The analysis was informed by performance theory^14,37^ and by the broader life narratives of each of the 15 women included in this study. A narrative performative analysis entails a focused analysis of the accounts and self-narrations of life events, ‘both as constructions and claims of identity’.^45^ We conducted a two-phased narrative performative analysis to explore the broader life stories and identify the specific performances (moments) of HIV status disclosure of each participant. In phase one, we collated data collected across all research modules for each of the 15 women to construct a cohesive case description of each participant, including their kinship maps, housing arrangements, economic prospects, romantic relationships, illness narratives and health access history. Data included verbatim translated transcripts of in-depth interviews, field notes, photos and detailed reflective notes. Second, we focused on the specific speech sections where participants described the act(s) of disclosing (or deliberately concealing) their HIV status to their sexual partners. We then identified overarching themes^46^ for the various types of performances of (non)disclosure. Through narrative analysis, we engage with several ‘lenses’ in considering the way that participants construct events, including language (word choices), the narrative process (sentence structures), context (in which events take place) and moments (identifying key events).^47^

From the analyses, we describe key ‘moments of disclosure’ narrated by participants during the extended course of data collection. We present these disclosure narratives with supporting longitudinal data from each participant where they described their intimate sexual relationships in detail.

Approval was provided by the London School of Hygiene and Tropical Medicine Ethics Committee, Stellenbosch University Research Ethics Committee, and the Bio-medical Research Ethics Committee at the University of Zambia (N12/09/056; N12/11/074). All participants signed written informed consent and continually reconfirmed over time. Pseudonyms are used throughout for reporting purposes.

## Findings

In our findings, we present data from 15 women aged 19–45 years, from 12 households. In terms of demographic representation, nine of the women were Black African, Xhosa-speaking, and six women were coloured, Afrikaans-speaking community members (see Table 1).(During the apartheid era in South Africa, persons were ranked and segregated by race with privilege and freedom allocated in line with these categories. Other researchers have noted that ‘although the notion of racial groups is now legal history, it is not always possible to gauge the effects of past discriminatory practices . . . without referring to race. For this reason, (we) use the terms “black African” and “coloured”. . . where pertinent to the data analysis’.^48^)

**Table 1.** Pseudonyms and demographic description of participants.

Pseudonym	Age	Language	Romantic partnership
Cynthia	33	Afrikaans	Steady relationship with Peter.
Lana	28	Afrikaans	New relationship with Simon.
Asanda	42	Xhosa	Divorced.
Chantelle	31	Afrikaans	New relationship with long-term friend, Bradley.
Ntombi	40	Xhosa	Steady relationship with Chikhu; casual relationship with Esihle.
Millie	45	Afrikaans	Married to James.
Mara	21	Xhosa	Steady relationship with Zonke.
Cebisa (daughter of Nosipho)	19	Xhosa	Single, previous relationship with Xola.
Nosipho (mother of Cebisa)	48	Xhosa	Divorced.
Rebecca	26	Xhosa	Previous relationship with Danny.
Abby (lives with Stella)	20	Afrikaans	Several casual sexual partners.
Tina (lives with Stella)	21	Xhosa	Long-term relationship, Ben.
Stella (lives with Tina, Abby)	35	Xhosa	Long-term partner, Thando.
Fezeka	45	Xhosa	Long-term partner, Fuzile.
Dora	30	Afrikaans	On/off relationship with Hector.

We found that, for several women, disclosure (or deliberate non-disclosure) served a purpose beyond relaying biomedical information to inform sexual practices or for accessing HIV treatment support. Disclosure narratives were constructed according to broader scripts specifically performed to convince the audience or co-actors (intimate partner in this case) of the woman’s intentions or position. We grouped these performances into three categories: cementing relationships, betrayal, and anger, and ensuring self-preservation.

### Cementing relationships: disclosure as affirmation of intimacy

For several women, HIV disclosure was positioned as a productive next step towards affirming their intimate relationship with their sexual partners. Dora (aged 30 years) was diagnosed by the PopART intervention team in 2015 and, with the support of her close-knit family, initiated early treatment (outside of national guidelines, which at the time required a CD4 count of less than 500 for eligibility) soon thereafter. When we spoke to her, Dora was in an on-again, off-again relationship with her neighbour, Hector (55). Dora described how Hector made her feel special and that he took good care of her: ‘he does everything for me . . . he is a good person’. For Dora, the act of disclosure was positioned around the intention of ‘being honest’ with her partner, and in so doing, cementing their relationship.

During one of our discussions with Dora she described how, on seeing PopART community health workers (CHWs) who conduct door-to-door HIV testing in her neighbourhood, she preemptively engaged in a performance to assess Hector’s reaction to the possibility that she might need health support. In this prelude, Dora positioned herself as vulnerable and in need of care. In her account, Hector’s initial response to potentially distressing news assured her of his preservation of their relational script casting him as supportive partner which led to her disclosing her HIV status later that day:

[Hector] asked me, ‘how’s it going?’ And, ‘are you okay?’ Then I said, ‘no, give me another hug’. Then [Hector] said, ‘it’s going to be fine’. And then he asked, ‘what’s it about?’ I said, ‘I’m going to talk with you later’ [pause] and then later I told him.

Although the preliminary conversation gave Dora sufficient confidence that Hector would react supportively to her HIV status disclosure, the excerpt below illustrates just how tenuous the moment of disclosure can be,

Researcher What did he say?

Dora He was upset and so on. Then I told him, if he’s not happy with me, then he must say ‘no’. I’ll take my things and leave, I’ll move [end the relationship]. Then he said, ‘no, it’s good’ I told him, and I was honest with him. And then he also said now [disclosed his HIV-positive status].

Researcher He also told you about himself [his HIV status]? [Dora nods] And how did you feel that he didn’t tell you about it earlier?

Dora I wasn’t angry. Now if I was negative, then I would’ve been angry. But because I’m now already positive [it is okay]. We sat and spoke now [pause, exhales] and after that time, then everything was okay again.

When asked why she had disclosed, Dora explained to us that they were ‘at that stage of their relationship’, indicating that, for her, status disclosure was expected when a relationship had reached a certain level of seriousness. Dora’s status disclosure was thus a carefully constructed performance, guided by the intention to strengthen their bond. When it seemed that Hector might be going ‘off-script’ and not reciprocating her investment in the relationship, Dora threatened to end the relationship. However, with Hector’s acceptance and unexpected co-disclosure of also being HIV-positive, the status of their relationship was reaffirmed. In addition, through Dora’s disclosure, she cast herself in the positive framing of an ‘honest’ partner. In this disclosure dance, the parties co-constructed a script that championed their relationship above whatever social and health-related issues that come with living with HIV. To note, according to her account, at no point during their mutual disclosure did Dora, or Hector, mention the health-related aspects of HIV (treatment and transmission) or benefits of having disclosed (adherence support).

While HIV disclosure is often carefully planned and scripted, as indicated above, reactions from partners that do not adhere to expected scripts are disruptive and threaten the discloser’s identity and relationship with their partner. Lana (aged 28 years), for instance, went through careful measures to conceal her HIV status from others, including hiding her ART in the ceiling of the home she shared with her group of friends. She was diagnosed with HIV by the PopART intervention CHWs in 2015. During our interview in 2016, Lana was in a new relationship with one of the men in her social group, Simon (aged 28 years). Lana told us that she had told Simon about her diagnosis because ‘I always trusted him, see, I could talk to him. Other people close to me aren’t like that’. However, when asked about Simon’s reaction, Lana described the following:

Researcher What did he say [about your status]?

Lana [extended pause] It is another story.

Researcher When did you tell him?

Lana When the two of us tested.

Researcher Were you and Simon together when you tested? [Lana nods] And so you tested positive [Lana nods] And he? [Lana nods]. So, you were positive already? [Lana nods] So you tested again with Simon?

Lana Yes, but then Simon was positive already [knew his status prior to this test].

Researcher Oh, he also knew? [Lana nods] And so what did he say− [Lana interrupts]

Lana −I did not know.

Researcher What happened then?

Lana Because I did not know about him [pause]. I just told him, but [pause, hesitant] I did not know about him [pause], and he is not one who freaks out [overreacts]. He is just calm, but I have not [pause, reconsiders]. He did not tell me about himself [exhales loudly, sounds reproachful].

Researcher And was he ugly [mean/rude] towards you?

Lana No, he [pause] he knew about himself.

Researcher He told you he knew already [about his own status]?

Lana No, he did not tell me. I found out at the clinic [inaudible]. I was getting angry, because I told him about me [my status] but he has not told me about himself [his status].

Lana described how she used the opportunity of retesting for HIV with the CHWs to inadvertently ‘reveal’ her status to Simon. When she tested positive, Lana explained, she told Simon that she had known she was living with HIV for a few months. Lana wanted to disclose to Simon because she thought of him as kind and trustworthy. Since Lana viewed Simon as supportive, she expected that her disclosure would be met with acceptance, even though he tested positive. Lana cast both herself and Simon as trustworthy. However, according to Lana, she inadvertently found out at the clinic that Simon had long been aware of his positive status, leaving her feeling betrayed. In this instance, the ‘disclosure script’ unfolded positively, and as expected, however the broader ‘relational script’ appears to have been fractured by Simon’s decision not to disclose his status earlier on in their relationship. While status disclosure presented a challenge to our participants’ relational scripts, it also revealed an opportunity to cement relationships especially when both partners were living with HIV. However, the disclosure process was, ultimately, influenced by the partner’s HIV status and their willingness reciprocates disclosure.

For others, like Chantelle (aged 31 years), HIV status disclosure was described as an affirmation of trust in her long-term childhood friend, neighbour and eventual boyfriend, Bradley (aged 27 years). Prior to her relationship with Bradley, Chantelle had been in several abusive relationships and had struggled with substance use. She described her disclosure to Bradley:

Before the two of us were bound [had sex], I already opened up to him. He is my friend and I sat with him. We share everything with each other, as friends. We help each other . . . I opened up to him. I told him what I’d been through. I spoke to him about myself, that I have HIV so he would know, as a friend. I don’t have other friends. But I didn’t know how he is going to be [react] and how he’s going to speak to me. Is he going to be upstairs [looking down on] towards me? Many *tjommies* [buddies] . . . when they hear that a friend is HIV-positive, then they move their company [friendship] away.

As in the previous disclosure narratives, Chantelle described the angst and uncertainty she felt about disclosing to Bradley. Unlike with other participants, Bradley maintained Chantelle’s script and turned the conversation towards a discussion on HIV transmission and safe sex. Chantelle explained,

Bradley said, ‘it’s not about being contagious. You just have to know how to avoid it [HIV transmission]. You won’t have clean [condom-less] sex, [you need to know what to do] if you have cuts’. Furthermore, the two of us, we understand each other. He understands me and I understand him. He accepts what I have been through.

Maintaining a script of trust and understanding and managing Chantelle’s identity in their larger social group led Bradley to procure condoms without raising suspicions of Chantelle’s HIV status. Together, the couple engaged in a wider social script:

Bradley goes to the clinic, or he asks his friends [for condoms]. Then they want to know, why he’s asking for condoms. Then he says, ‘it’s for CD discs, to clean them’. [laughing] He tells them . . . you get the oil from the condoms and you rub it on [the cd]. He [tells them]: you can put it on your skin too. ‘I need those things [condoms] for that [cleaning CDs, skin lotion], not for women [sex], I mean, do you know me?’

For Chantelle, her status disclosure was meant to cement her relationship with Bradley. When Bradley also engaged in an elaborate act to conceal Chantelle’s HIV status from their friends, we see how the couples’ script bleeds into a wider script they both ascribe to in their social group. In this wider social script implied by Bradley’s rhetorical adage (‘I mean, do you know me?’), safe sex (and HIV by implication) is positioned as outside of the norm for their group. Although the initial disclosure led to a discussion on safe sex practices, the logico-scientific information served to carve out a script both parties could ascribe to – one where HIV is not part of their socially performed personas – rather than being the centre around which other scripts revolve. It is important to note that neither Chantelle nor Bradley were aware of ART as a form of HIV prevention, despite living in a community where universal access to ART and door-to-door HIV testing was available.

### Betrayal and anger

For other women, the act of disclosure was the aftermath of an unanticipated HIV-positive diagnosis. For these women, disclosure was part of a process of confrontation and acknowledgement of physical and emotional betrayal by their partners. In the moment of an unexpected HIV diagnosis, existing relational scripts came undone. This follows Bury’s observations that an unexpected illness diagnosis ‘brings individuals, their families, and wider social networks face to face with the character of their relationships’.^23^ By disclosing their HIV status, some participants articulated a break in trust and disruption in their relationships, while others used the opportunity to force their partners to acknowledge infidelity and disclose their own HIV-positive status in an attempt to stitch the threads of their relationships together. Where the preexisting script was irreversibly spoilt, the women had to reevaluate their relationships and create new scripts based on new self-identities and impressions of their partners, shaped by the unwelcome introduction of HIV into their relationship.

Millie (aged 45 years) had been married to James (aged 47 years) for several years. She described her relationship with James as tumultuous – with James often drinking too much alcohol, taking drugs and disappearing for days at a time. However, Millie also describes herself as being deeply in love, and, every time James returned home, she would allow him to stay. Millie explained that she had unexpectedly tested positive for HIV during a routine household visit by the PopART intervention CHWs. James was out at the time, but her three children (from her previous marriage) were at home. The diagnosis was especially difficult for Millie, she explained because she was deeply religious. After reflecting on her diagnosis, she suggested that it must have been God who sent the CHWs so that she could become aware of her status and start medication. However, it was difficult for her to reconcile the romantic and emotional bonds she shared with James, with the realization that he had betrayed her and that he had been unfaithful. She recounted how she confronted James about her HIV status, conveying her anger, betrayal and shock:

Millie It was tense [emphasis]. He walked out, then he came back the night again.

Researcher Did he then get in bed? [Millie confirms]. How did you feel that time? No anger, no emotions?

Millie Maybe.

Researcher We− [Millie interrupts]

Millie −maybe in my insides, sore, heartbroken and such things. [Whispering] I didn’t expect something like that, that it would happen. It also came back to me. Okay so maybe that was what [he] wanted to talk to me about. [He] said there is something that [he] wanted to tell me, but [he] didn’t know how I would accept it.

Researcher So he had already wanted to tell you something? Do you think he knew about it [his positive status]?

Millie Maybe. Yes, but he never talked about it . . . I said to him, ‘you gave it to me’. I said, I just said, ‘ok, fine it is how it is’.

She continued to explain her shock and disappointment and how disclosure led to exposing other issues in the relationship:

I wasn’t expecting it you know. But, as the time passes [you think], why would someone you love, you really love [stops mid-sentence]. That’s why I was saying, if he loves me, why would he hurt me in this way, you know? I told him, ‘tell me, if you feel you don’t want to be with me, because, I can talk to you openly about how I feel’. But he said I won’t understand him because we won’t be speaking the same language. So, I asked him, ‘just tell me if you don’t want to be in my life anymore, [if you] can’t take this’. He didn’t want to talk. He said I won’t understand him. I said, ‘so what does this mean? Where does this end? So, you think this must go on and on and on and I must just be at ease with you coming and going?’

Millie description shows how the preexisting scripts where the couple was cast as committed, loyal and loving, had been ruined by James. Despite this, she attempted to recreate a new script in which she will no longer ‘be at ease with [James] coming and going’. Although Millie positions herself as subject to James’ choice to take the lead in ending the relationship (to tell her if *he* doesn’t want to be with her), she also sketches new boundaries in which their relationship must exist. In Millie and James’ relationship, the act of disclosure was not associated with accessing treatment support or supporting other health-related decisions, but rather, the conversation was used as a way for Millie to recast a script in which underlying issues in their relationship would be averted and in which trust could, perhaps, be reestablished.

Rebecca (aged 26 years) is a shy woman who lives in a small informal home with 10 other extended family members and friends. When she was in high school, she had a sexual relationship with a taxi driver in his late 20s. Although occurring long after the relationship ended, she confronted him after being diagnosed with HIV:

He was a man driving a taxi, I was still a school child. I used to go to him just for sex, then come back here at home again. When I was breaking up with him, he called. I didn’t know that I was sick, he called saying, ‘do you know that you are sick?’ But I never took that serious. I was still dumb; I didn’t ask him. So, after years I just fell sick. I had TB and my mind came back to that first guy that I used to date. I called him back. Of course, when I called him, I insulted him, told him that he is cruel for not telling me that he is sick, for not putting on a condom and so on. For not wanting to go to a clinic [to test], you see. So, he said that he doesn’t know about that, and I told him that he should stop denying it.

In telling Rebecca that she is ‘sick’, the taxi driver engaged in a nonchalant disclosure performance. For Rebecca, the former boyfriend is the target of her anger, but the betrayal she experienced at her diagnosis appeared to be directed towards herself. That is, she appears to rebuke herself for being naïve and ‘dumb’. By confronting the taxi driver, Rebecca appears to be recreating a script and identity in which she is wiser and regains agency, up to where she is able to reprimand the older man to ‘stop denying’ that he has HIV. Rebecca’s act of disclosure conveyed several intentions. While Millie and Rebecca’s anger towards their intimate partner was partly related to the impact of acquiring an incurable condition, it was the implicit betrayal that was being relayed. For these women, an HIV diagnosis signified that their partner was either dishonest or deceitful and the act of disclosure was a means to elicit a confession or to highlight other shortcomings in the relationship.

### Ensuring self-preservation

For some women, not disclosing their HIV status to their intimate partner was a calculated decision. To conceal their HIV status, these women presented particular performances, aimed at maintaining the boundaries of privacy (or the dramaturgical backstage). For instance, Cebisa (aged 19 years), who became HIV-positive through vertical transmission, chose not to disclose her status to her boyfriend as an act of self-preservation. She described to us how she had been on HIV treatment ‘her entire life’, but only started questioning her mother about the medication when she was about 12 years old. Her mother was her main source of health support, as both her brothers were HIV negative – one born before her mother contracted HIV and the other after treatment for pregnant women became available. Cebisa has disclosed her HIV status to only one close friend, whom she describes as caring and loyal. She conceals her treatment and her diagnosis from everyone else. Cebisa spoke about her first serious boyfriend, Xola, whom she met in school when she was 16 and he was 18. She explained her feelings towards him:

Cebisa Yho! [Exclamation]. The problem is, I loved him very much

Researcher What is it you loved about him?

Cebisa Yho! Even him, the way he was [Cebisa smiling], the way he was speaking, everything about him I loved it [laughs]. He was a right/good person [smiling].

Cebisa explained that she had a sexual relationship with Xola, and how she negotiated condom use without revealing her status:

Researcher So sometimes you would use protection sometimes you would not use a protection?

Cebisa No, I never used it when I first slept [had sex] with him [brief pause], because I didn’t know what to say. I wanted protection for him because he trusted me. I also trusted him but then now I deceived him. I said that I don’t inject the needle [contraceptive injection]. So [inhales loudly], we must use condoms, then he agreed.

Researcher So then would he agree to have a condom? [Cebisa nods] You were on [family] planning at the time?

Cebisa Yes, I was on [family] planning

Cebisa explained that she cared for her partner, and to protect him, she orchestrated the scene where condom use was presented as a way of preventing pregnancy, not HIV transmission. For Cebisa, condom use for pregnancy prevention, despite already using another form of pregnancy prevention (Depo-Provera injection), was a more acceptable scripted performance than revealing that she was living with HIV. This alternative script is exemplified by Cebisa’s admitting to ‘deceiving’ Xola. Her non-disclosure is purposefully orchestrated as self-preservation, in addition to the perceived preservation of the relationship:

Researcher Do you ever say, look this is what is happening and disclose [your status]?

Cebisa No never. [I] don’t do that thing, I have never done it [disclosed].

Researcher Why?

Cebisa No, yho [exclamation, laughing]

Researcher Why Cebisa?

Cebisa No.

Researcher Why, [are] you afraid?

Cebisa Yes, I’m afraid.

Researcher What are you afraid [of]? That he would dump you? He would beat you?

Cebisa Yes, I’m afraid [of] those things, maybe he dumps me.

The couple dated for 2 years, but Cebisa ended the relationship after she heard that he had ‘cheated on her’. Somewhat ironically, Cebisa explained that trust in relationships is very important to her and that you cannot have two girls from the same school dating the same boy.

Ntombi (aged 40 years) became sick in 2014 and was diagnosed with HIV at the clinic. She started on ART in 2015 as part of the PopART trial, prior to the changes in HIV treatment national guidelines. When asked why she decided to access ART, she explained that she did not want to ‘be behind’ and continued: ‘I wanted to know where I stand. I must know myself’. When we spoke to Ntombi, she was in a relationship and living with Chikhu. The couple shared Chikhu’s house, and Ntombi ran a small business selling alcohol from their home. Although Ntombi was fond of Chikhu, she complained that he no longer satisfied her sexually, and that she had another casual partner (Esihle) that she met at a tavern. Neither of these men were aware of Ntombi’s HIV status. When asked why she did not want to disclose to Chikhu, Ntombi explained:

Ntombi My reasons for me not wanting him to know is that, I see that he is rude. And he is as rude as he is proud. He is proud. Too much. He is full of himself. And he doesn’t take nonsense, you see? When a person loves, [they] will be there. Even if it means to die with you. But him, no.

Researcher This guy?

Ntombi Yes. It is about three times now, [he was] throwing me out of the house. I also told myself that no, there’s no need to show him who I am. Let me just keep it this way and continue the way we are. Because then from the very start when I met him, we used a condom. And he doesn’t take a risk. If it’s not there, it’s not there, simple [meaning, no sex without a condom].

Ntombi emphasizes that she hides her true self from Chikhu. Ntombi’s internal script of what it means ‘to love someone’, to ‘be there even if it means to die with you’, has been violated on at least three occasions by Chikhu, without her disclosing her HIV status. As such, she performs according to alternative script both to conceal ‘who she is’ and to protect herself from a potentially ‘rude’ reaction from Chikhu. Ntombi described her relationship with Esihle as casual, although she did start having more serious feelings towards him at one stage. She told us that she enjoyed his company, and they were having sex, but, when we last spoke to her, she did not foresee a long-term relationship. Ntombi employed several metaphors to explain why she did not disclose her status to him:

When you see it’s too windy, you leave. So, before you do that thing, you have to see first that it is okay. If I pour sugar, how sweet will this sugar taste? I realised, okay fine. We are in love. Everything is nice the way things are happening. I don’t want to tell him. Let’s just keep it this way. If we are already using condoms, we are right on that side. Because I don’t know what will happen in the end.

Ntombi provided three reasons for her non-disclosure: disclosure might be disruptive for the relationship (cause wind/turbulence); if she discloses, the consequences are unknown (adding sugar will alter the taste); and as they were already using condoms, there was no need to disclose. For Ntombi, fear of the unknown consequences of disclosure was presented as the reason for non-disclosure. Non-disclosure is also seen as a justifiable performance ( ‘on the right side’) as she insists on using condoms. In addition, Ntombi also appears to be relatively powerless in her relationship with Chikhu, on whom she relies for a place to stay and run her shop. She does not foresee any possibility of creating a script in which Chikhu reacts in a supportive way following disclosure. An unsure future with Esihle meant that Ntombi decided not to disclose her status to him either. However, it appears Ntombi is fully aware of the risks of HIV and has managed to both refrain from unprotected sex and maintain a script in which her HIV status goes unquestioned by her partners.

## Discussion and conclusion

We found that HIV status disclosure (and deliberate non-disclosure) is part of a carefully scripted performance, where the end product is not the sharing of biomedical information but rather to communicate underlying social intentions and to affirm social- and self-identities. Some women disclosed their HIV status to convey their trust in their partners and to cement their relationships. In preparing for disclosing, these women anticipated certain positive (scripted) reactions from their partners – acceptance, support and understanding. However, as we show, partner performances were at times discordant with women’s expectations, leading to tension in relationships. For others, disclosure was in reaction to receiving an (mostly unanticipated) HIV-positive diagnosis. For these women, disclosure was instrumental in conveying anger and betrayal. More than blaming their partners for transmitting a sexual infection, these women wanted to express their anger/anguish because of the breakdown of the relationship and the partner’s assumed sexual transgressions. Other women participated in different performances to avoid telling their partners about their HIV status, either as a form of self-preservation or for the preservation of the relationship. Performances included engaging in more acceptable social scripts and acts, such as condoms for pregnancy prevention, rather than HIV prevention. In disclosing, women engaged in (re)constructing their identities as PLHIV in intimate relationships – either as good/honest partners strengthening relationships; as betrayed partners reevaluating relationships or as women who were claiming agency and ensuring self-preservation. HIV prevention, in general, and treatment adherence support overall, was rarely mentioned as a reason for disclosure – and never as the primary driver for HIV disclosure. At the time of our research, the message of HIV treatment as form of prevention had not yet filtered down into the intimate disclosure narratives of women who had access of treatment regardless of CD4 count. It is plausible that scale-up of UTT has, to date, missed an opportunity to support disclosure thereby impeding the potential patient and public health benefits of this approach.

Similar to other research focusing on HIV disclosure practices, we highlight that the process of revealing one’s HIV status is simultaneously a test of intimate relationships and a reconciling of self-identity.^42^ Bury noted that acquiring an illness diagnosis, and in effect, decisions around disclosure are ‘disruptions in explanatory systems normally used by people, such that a fundamental re-thinking of the person’s biography and self-concept is involved’.^23^ We suggest that, in selected (non-)disclosures, women living with HIV try to maintain their relationship scripts with the intention of avoiding further disruptions to their lives. Wekesa and Coast^49^ noted that ‘incorporating HIV . . . into one’s identity also involves multiple phases of identity transition, including diagnosis, (non-)disclosure, positive living and attempts at repair and normalcy’. Others have suggested that considerations of disclosure are driven by potential social gains in support or evasion of negative reactions.^41^ In highlighting the complexities of disclosure, it is worth noting that, while public health messaging might be focused on HIV prevention and treatment adherence, HIV is not a standalone condition but is often nested in the complexities of intimate relationships.^50^

By drawing on rich longitudinal data set, we were able to support accounts of disclosure with additional longitudinal data on intimate relationships.(For a detailed description of the data collection process, see Viljoen, Myburgh, and Reynolds (2020)^21^). As an additional strength, the analyses were informed by the experiences of the primary authors who were involved in the conceptualization, design and data collection of the project and received input from an experienced multi-disciplinary research team familiar with the HIV landscape in South Africa.

As a limiting factor, we were only able to analyze the disclosure narratives presented to us by participants. As such, we present an analysis of the retelling of women’s performances, rather than the disclosure performance itself. Participants could also have viewed the researchers as another type of audience, where retelling their disclosure stories allowed them to ‘construct themselves and others as particular kinds of moral agents’.^51^ However, through longitudinal data collection, we are able to supplement once-off narratives of disclosure with broader and repeated life narratives and in doing so, present comprehensive and informed accounts.

We have shown that HIV status disclosure happens in context of tensions and overlapping priorities, such as relationship dynamics and identity formation. In addition, there are apparent competing disclosure principles: public health systems are focused on treatment adherence and transmission prevention while women living with HIV are focused on the dynamics of interpersonal relationships. In addition to other stresses in high-burden, low-resource communities, PLHIV often have challenges in preserving confidentiality, as the decisions around disclosure are complicated by a lack of private spaces.^21^ It is in these spaces where effective HIV counselling could be instrumental in facilitating disclosure processes by simultaneously providing interpersonal relationship support and engaging couples in the personal and public health benefits of ART adherence.
